# The Aquaporin Splice Variant *Nb*XIP1;1α Is Permeable to Boric Acid and Is Phosphorylated in the N-terminal Domain

**DOI:** 10.3389/fpls.2016.00862

**Published:** 2016-06-16

**Authors:** Henry Ampah-Korsah, Hanna I. Anderberg, Angelica Engfors, Andreas Kirscht, Kristina Norden, Sven Kjellstrom, Per Kjellbom, Urban Johanson

**Affiliations:** Center for Molecular Protein Science, Department of Biochemistry and Structural Biology, Lund UniversityLund, Sweden

**Keywords:** XIP, AQP, NIP, *Nicotiana benthamiana*, boric acid, phosphorylation, *Pichia pastoris*

## Abstract

Aquaporins (AQPs) are membrane channel proteins that transport water and uncharged solutes across different membranes in organisms in all kingdoms of life. In plants, the AQPs can be divided into seven different subfamilies and five of these are present in higher plants. The most recently characterized of these subfamilies is the XIP subfamily, which is found in most dicots but not in monocots. In this article, we present data on two different splice variants (α and β) of *Nb*XIP1;1 from *Nicotiana benthamiana*. We describe the heterologous expression of *Nb*XIP1;1α and β in the yeast *Pichia pastoris*, the subcellular localization of the protein in this system and the purification of the *Nb*XIP1;1α protein. Furthermore, we investigated the functionality and the substrate specificity of the protein by stopped-flow spectrometry in *P. pastoris* spheroplasts and with the protein reconstituted in proteoliposomes. The phosphorylation status of the protein and localization of the phosphorylated amino acids were verified by mass spectrometry. Our results show that *Nb*XIP1;1α is located in the plasma membrane when expressed in *P. pastoris*, that it is not permeable to water but to boric acid and that the protein is phosphorylated at several amino acids in the N-terminal cytoplasmic domain of the protein. A growth assay showed that the yeast cells expressing the N-terminally His-tagged *Nb*XIP1;1α were more sensitive to boric acid as compared to the cells expressing the C-terminally His-tagged isoform. This might suggest that the N-terminal His-tag functionally mimics the phosphorylation of the N-terminal domain and that the N-terminal domain is involved in gating of the channel.

## Introduction

Aquaporins (AQPs), also referred to as Major Intrinsic Proteins, facilitate the transport of water and/or other small uncharged molecules across membranes in all kingdoms of life (Gomes et al., 2009). AQPs primarily exist as homotetramers in which each monomer has six transmembrane helices and five loops with both the N and the C terminus located in the cytoplasm. Two of the loops form half-helices that contain the highly conserved AQP asparagine-proline-alanine (NPA) motif at their N termini. The two short α-helices dip into the membrane from opposite sides and the N termini, with the NPA boxes, meet in the middle of the pore creating a seventh transmembrane structural unit, in addition to the six membrane spanning α-helices.

In mammals, 13 AQP isoforms have been identified, which are involved in various physiological functions (Agre and Kozono, 2003). In plants, however, more than 13 AQP isoforms have been identified; for instance, 35 in *Arabidopsis*, 36 in maize, 33 in rice, 55 in poplar, and 71 in cotton (Chaumont et al., 2001; Johanson et al., 2001; Sakurai et al., 2005; Gupta and Sankararamakrishnan, 2009; Park et al., 2010). Based on sequence similarity, AQPs of higher plants are divided into five subfamilies namely: the Plasma Membrane Intrinsic Proteins (PIPs), the Tonoplast Intrinsic Proteins (TIPs), the Nodulin-26 like Intrinsic Proteins (NIPs), the Small basic Intrinsic Proteins (SIPs), and the X Intrinsic Proteins (XIPs; Johanson et al., 2001; Danielson and Johanson, 2008; Bienert et al., 2011). Interestingly, XIPs which were first discovered in the moss *Physcomitrella patens* (Danielson and Johanson, 2008) have not only been identified in plants such as grapevine (*Vitis vinifera*; Danielson and Johanson, 2008), poplar (*Populus trichocarpa*; Gupta and Sankararamakrishnan, 2009), tomato (*Solanum lycopersicum*; Sade et al., 2009; Reuscher et al., 2013), cotton (*Gossypium hirsutum*; Park et al., 2010), tobacco (*Nicotiana tabacum*; Bienert et al., 2011), and bean (*Phaseolus vulgaris*; Ariani and Gepts, 2015) but also in fungi (Gupta and Sankararamakrishnan, 2009) and protozoa (Danielson and Johanson, 2008). Based on phylogenetic analysis, XIPs of higher plants have been grouped into two distinct clusters termed XIP-A and XIP-B, where XIP-A includes only a XIP1 subgroup and XIP-B is divided into four subgroups, i.e., XIP2, XIP3, XIP4, and XIP5 (Lopez et al., 2012). According to this classification *Solanales* XIPs including the XIPs from *N. tabacum*, *N. benthamiana*, *Ipomoea nil*, and *S. lycopersicum* were assigned to subgroup XIP4 (Lopez et al., 2012), while XIPs in rubber tree (*Hevea brasiliensis*), castor bean (*Ricinus communis*), physic nut (*Jatropha curcas*) and poplar (*P. trichocarpa*) have been assigned to subgroups XIP1, XIP2, and XIP3 (Zou et al., 2015a,b, 2016). In a more recent phylogenetic classification of XIPs of land plants only four groups (XIP I-IV) are proposed (Venkatesh et al., 2015). In this classification, subgroup XIP IV is corresponding to group XIP4. For simplicity the original naming of the *Solanaceae* XIPs as XIP1s has been retained as it is also used in the work of Venkatesh et al. (2015). In *Solanaceae* the pre-mRNAs of some *XIP1* genes undergo alternative splicing resulting in two variants of the transcript and thus in two slightly different proteins (Bienert et al., 2011). *XIP1;1* mRNAs were found to be expressed in all organs of the tobacco plant and the proteins were localized in the plasma membrane of plant cells (Bienert et al., 2011). Transiently expressed *Solanaceae* XIP1;1s facilitated the transport of glycerol but not water in *Xenopus oocytes* and when expressed in *Saccharomyces cerevisiae* mutants increased the sensitivity of the cells to boric acid, urea, and hydrogen peroxide (Bienert et al., 2011), which suggests that XIP1;1s are not primarily water transporters (Bienert et al., 2011; Lopez et al., 2012). However, the physiological function and the *in vivo* substrate of XIPs are yet to be discerned. It is therefore necessary to characterize XIPs further with regard to selectivity, structure, and regulation.

The opening and closing of the pore of AQPs, often referred to as AQP gating, have been observed for mammalian, plant, and yeast AQPs (Törnroth-Horsefield et al., 2006; Fischer et al., 2009). Divalent cations, pH and phosphorylation have been reported to regulate AQP gating and water permeability (Maurel et al., 1995; Johansson et al., 2000; Guenther et al., 2003; Daniels and Yeager, 2005; Temmei et al., 2005; Törnroth-Horsefield et al., 2006; Azad et al., 2008). Phosphorylation increased the water channel activity of *Pv*TIP3;1 (Maurel et al., 1995), *So*PIP2;1 (PM28A; Johansson et al., 1998), soybean nodulin 26 (Guenther et al., 2003), and *Tg*PIP2:2 (Azad et al., 2008). The *So*PIP2;1 in *Spinacia oleracea* was shown to be regulated by phosphorylation at Ser 115 and Ser 274 (Johansson et al., 1998; Törnroth-Horsefield et al., 2006). Likewise, the soybean nodulin 26 was reported to be phosphorylated at Ser 262 (Lee et al., 1995; Guenther et al., 2003).

For crystallization and structural determination, not only large amounts of proteins are required but also homogenous samples are essential for successful crystallization. *Pichia pastoris* as expression system has been shown to generate high yields of functional AQPs (Karlsson et al., 2003; Horsefield et al., 2008; Nordén et al., 2011) and *P. pastoris* has the machinery to phosphorylate heterologously expressed eukaryotic proteins (Hori et al., 2010). Phosphorylation of plant AQPs in *P. pastoris* has been shown (Daniels and Yeager, 2005) and this correlates with results from mutant studies and from studies involving chemical inhibition of kinases and phosphatases (Azad et al., 2008).

In this study, we present results concerning the expression, purification and characterization of *N. benthamiana* XIP1;1 splice variants in *P. pastoris* and demonstrate phosphorylation of *Nb*XIP1;1α in its N-terminal region.

## Materials and Methods

### Cloning and Generation of Multi-copy *P. pastoris* Transformants

The cDNA encoding *Nb*XIP1;1α and *Nb*XIP1;1β (Nb_Ea0003A8 and Nb_Ea0006N9, Arizona Genomics Institute) were amplified by PCR, generating either N- or C- terminally His-tagged constructs with the following primer pairs: For N-terminally His-tagged constructs, TEV*Eco*RI-XIPfw (5′-TT**GAATTC**CGACCGAAAACCTGTATTTTCAGGGCATGTCTGCTTCCAATACTAGTCA-3′) and TEV*Not*I-XIPrev (5′-TT**GCGGCCGC**TCATACATGCAACCCAAATGAAG-3′); and for C-terminally His-tagged constructs, *Eco*RI-XIPfw (5′-CG**GAATTC**AAAATGTCTGCTTCCAATACTAGTCA-3′) and *Not*I-XIPrev (5′-TT**GCGGCCGC**TACATGCAACCCAAATGA-3′) where *Eco*RI and *Not*I restriction sites are indicated in bold and *P. pastoris* consensus start sequence are underscored. The PCR products were cloned into a modified pPICZB vector (Invitrogen) conferring the addition of a His_10_-tag and a TEV protease cleavage site to the N-terminus or the unmodified plasmid adding a myc antibody epitope and a His_6_-tag to the C-terminus of the amino acid sequence. The resulting plasmids were sequenced and transformed into wild type X-33 or protease deficient SMD1168H *P. pastoris* cells by electroporation according to the EasySelect− *Pichia* Expression Kit manual (Invitrogen, 2005). To obtain clones with potentially high copy numbers of the *Nb*XIP1;1α or *Nb*XIP1;1β splice forms, a zeocin selection was performed as described previously (Nordén et al., 2011). Briefly, transformed *P. pastoris* cells were plated onto YPDS [1% (w/v) yeast extract, 1% (w/v) peptone, 2% (w/v) dextrose, 1 M sorbitol] agar plates containing 100 μg/mL zeocin. The resulting colonies were then resuspended and the transformants were spread on plates containing higher zeocin concentrations. The plates were incubated at 28°C for 3 days and colonies were restreaked to obtain *P. pastoris* clones derived from single cells.

### Small-scale Expression Screen

A small scale expression screen was performed in order to analyze *Nb*XIP1;1α and *Nb*XIP1;1β expression capacity of X-33 and SMD1168H clones selected at the different antibiotic levels. Clones were cultured in 5 mL BMGY [1% (w/v) yeast extract, 2% (w/v) peptone, 100 mM potassium phosphate pH 6.0, 1.34% (w/v) yeast nitrogen base, 4 × 10^–5^% (w/v) biotin, 1% (v/v) glycerol] over night to generate biomass. Cells were harvested and resuspended in 5 mL BMMY [1% (w/v) yeast extract, 2% (w/v) peptone, 100 mM potassium phosphate pH 6.0, 1.34% (w/v) yeast nitrogen base, 4 × 10^–5^% (w/v) biotin, 0.5% (v/v) methanol] to an optical density at 600 nm (OD_600_) of 1. To sustain induction, methanol was added to a final concentration of 0.5% (v/v) every 24 h. Cell cultures were incubated with continuous agitation at 245 rpm for 72 h at 28°C. Cells corresponding to 20 OD_600_ units were harvested and disrupted, by vigorous vortexing with glass beads, in cold breaking buffer [50 mM NaPO_4_ pH 7.4, 1 mM EDTA, 5% (v/v) glycerol, 1 mM PMSF]. The lysate was cleared by centrifugation and the supernatant, containing the crude cell extracts was analyzed for *Nb*XIP1;1 content by western blot as described below. Since recombinant expression was more efficient in wild type cells, X-33 *P. pastoris* clones were used in further trials.

### Western Blot Analysis

To the crude cell extracts, 3.33 × SDS loading buffer [250 mM Tris-HCl pH 6.8, 40% (v/v) glycerol, 8% (w/v) SDS, 2.4 M β-mercaptoethanol, 0.1% (w/v) bromophenol blue] was added. Samples were solubilized at ambient temperature for 30 min. Proteins were separated on 12% SDS-PAGE gels and transferred onto polyvinylidene difluoride (PVDF) membranes (Millipore). His-tagged *Nb*XIP1;1 protein was visualized by probing with primary antibody mouse anti-(His)_4_ (Qiagen) and secondary antibody horseradish peroxidase-conjugated polyclonal goat anti-mouse IgG. Blots were developed by enhanced chemiluminescence.

### Yeast Growth Assay

To verify the functionality of the heterologously expressed *Nb*XIP1;1 protein, a yeast growth assay was performed. The X-33-XIPαHis_6_ clone, the X-33*-*His_10_XIPα clone, the X-33-His_10_NIP5;1 clone and a X-33 clone transformed with an empty pPICZB vector were cultured in small scale, as described above, and methanol induced protein expression was sustained for 26 h. The cell density was subsequently measured and the cultures were diluted to OD_600_ of 1. The induced *P. pastoris* cells were spotted onto YPD agar plates without boric acid and plates with 10 mM boric acid in a 1:10 dilution series. The plates were incubated at 28°C and after 5 days differences in growth were recorded.

### Stopped-Flow Spectrometric Assay of *P. pastoris* Spheroplasts

To test for water or glycerol permeability in the transformed cells, *P. pastoris* spheroplasts were prepared as published previously (Fischer et al., 2009) with slight modifications. In brief, *Nb*XIP1;1α or *At*NIP5;1 protein production was induced in transformed *P. pastoris* cells in BMMY with a starting OD_600_ of 1, as described above. After 26 h of induction, the cells were harvested, resuspended and incubated in TE-buffer (100 mM Tris-HCl pH 8.0, 1 mM EDTA) and 0.5% β-mercaptoethanol for 1.5 h to destabilize the cell wall. The cells were then washed and equilibrated in 20 mM MES pH 6.5, 1.2 M sorbitol with a final OD_600_ of 5 for 2 h. For water transport, equilibrated spheroplasts were subjected to a hyperosmolar solution (20 mM MES pH 6.5, with 1.8 M sorbitol) and the shrinkage upon mixture was observed by light scattering at 90° angle in a stopped-flow apparatus (SF-61 DX2 Double Mixing Stopped-flow System, Hi-Tech Scientific) at 500 nm. For glycerol transport, the equilibrated spheroplasts in 20 mM MES pH 6.5, 1.2 M sorbitol were mixed with equal volume of a hyperosmolar solution containing 20 mM MES pH 6.5 and 1.8 M glycerol and the initial shrinkage and subsequent swelling upon mixture were observed by light scattering at 90° angle in a stopped-flow apparatus at 500 nm. Kinetic Studio version 2.28 (TgK Scientific Limited) was used to calculate the rate constants and the standard error of estimate (SEE) of the traces.

### Isolation of *P. pastoris* Plasma Membrane

Plasma membranes were isolated from *P. pastoris* clones as previously described (Grillitsch et al., 2014) with slight modifications. Briefly, 20 g cell wet weight of *P. pastoris* cells were lysed by 8 × 30 s runs with 30 s intervening cooling sessions in a breaking buffer (25 mM Tris-HCl pH 8.5, 1 mM EDTA, 1 mM PMSF) by means of a BeadBeater apparatus (BioSpec Products). The cell lysate was centrifuged (1,000 ×*g*, 10 min, 4°C) to sediment unbroken cells. Bulk membranes were pelleted by centrifugation (35,000 ×*g*, 30 min, 4°C) from the supernatant. The pelleted bulk membranes were homogenized with 10 strokes using a hand homogenizer in TEDG-buffer [20% (v/v) glycerol in 10 mM Tris-HCl pH 7.5, 0.2 mM EDTA, 0.2 mM DTT] and the membrane suspension subsequently loaded onto a discontinuous sucrose density gradient consisting of 1:1 volume ratio of 43 and 53% (w/v) sucrose in TED-buffer (10 mM Tris-HCl pH 7.5, 0.2 mM EDTA, 0.2 mM DTT). Crude plasma membranes were collected from the gradient at the 43/53% sucrose interface after centrifugation in a swing-out rotor (SW 28, Beckman coulter) at 100,000 ×*g*, 4°C for 4 h. The crude plasma membranes were diluted threefold in ice-cold water and spun at 45,000 ×*g*, 4°C for 20 min in a JA-25.50 Beckman coulter rotor. The resulting pellet was homogenized with 10 strokes in MES-buffer (5 mM MES pH 6.0, 0.2 mM EDTA). Subsequently, the crude plasma membranes were loaded onto a second discontinuous sucrose density gradient consisting of 53, 43, and 38% (w/v) sucrose in 1:1:1 volume ratio in MES-buffer and centrifuged for 2.5 h at 100,000 ×*g*, 4°C. After centrifugation, the purified plasma membranes were collected at the 43/53% sucrose interface, diluted in Tris buffer (10 mM Tris-HCl pH 7.4) and pelleted as earlier described. Finally, the sedimented plasma membranes were homogenized in the Tris buffer and analyzed on a western blot for the presence of the His-tagged *Nb*XIP1;1 protein. The remainder of the plasma membranes were stored at –80°C.

### Large Scale Expression for Purification

Two clones of *Nb*XIP1;1α (designated X-33-XIPαHis_6_ and X-33His_10_XIP1;1α) and one of *Nb*XIP1;1β (designated X-33-XIPβHis_6_) with the highest protein expression levels, were used for purification trials. The X-33His_10_XIP1;1α clone gave the highest yield of purified *Nb*XIP1;1α protein and therefore was chosen for further purification and downstream application. Due to the benefits the controlled growth in bioreactors confer (Nyblom et al., 2007), the X-33His_10_XIP1;1α clone was eventually cultured in large scale using a 3 L bench top fermenter (Belach Bioteknik). A 100 mL X-33His_10_XIP1;1α *P. pastoris* pre-culture in BMGY was incubated at 30°C and 250 rpm over night to OD_600_ ∼10. The BMGY culture was, together with 6.5 mL PTM_1_ salts (Bushell et al., 2003), added to 1.5 L basal salt medium (Stratton et al., 1998), pre-tempered to 30°C in the fermenter. When the initial glycerol was consumed after approximately 24 h, a feed with 50% (v/v) glycerol was initiated to increase biomass. After 6 h, 200 mL of glycerol had been consumed and the culture had reached an OD_600_ of 210. To induce expression of *Nb*XIP1;1α, 100% methanol with 1.2% (w/v) PTM_1_ salts was added to the *P. pastoris* culture at a steady feed rate. Induction was sustained for 50 h after which 390 mL methanol had been consumed and the culture had reached an OD_600_ of 300. Cells were harvested by centrifugation and stored at –80°C until further use.

### Membrane Preparation

Cells corresponding to a wet weight of 40 g were thawed and resuspended in 150 mL ice-cold breaking buffer supplemented with complete Ultra protease inhibitor cocktail tablets (Roche). The cell suspension was, together with 200 mL cold glass beads, transferred to a BeadBeater (BioSpec Products) container. The cells were mechanically resuspended by 12 × 30 s runs with intervening 30 s cooling sessions. Unbroken cells and cell debris were removed by centrifugation at 1,400 ×*g* at 4°C for 30 min. Crude membrane fraction was subsequently collected by ultracentrifugation of the 1,400 ×*g* supernatant at 186,000 ×*g* at 4°C for 2 h and the resulting membranes were homogenized in cold buffer A [20 mM HEPES-NaOH pH 8.0, 50 mM NaCl, 10% (v/v) glycerol, 2 mM β-mercaptoethanol]. In order to remove peripherally bound membrane proteins and render the membranes more easily solubilized, a urea/alkali membrane wash procedure (Kistler et al., 1994; Fotiadis et al., 2001) was evaluated. However, since the 20 mM NaOH solution discharged part of the *Nb*XIP1;1 protein from the membrane, only the urea wash was subsequently applied. Briefly, 100 mL cold urea solution (4 M urea, 5 mM Tris-HCl pH 9.5, 2 mM EDTA, 2 mM EGTA) was added to crude membranes with a total membrane protein content of approximately 200 mg, and the mixture was incubated on ice for 30 min. The stripped membranes were sedimented by centrifugation at 186,000 ×*g* for 1.5 h at 4°C. Sedimented stripped membranes were resuspended and diluted ten-fold in cold buffer A. Finally, washed stripped membranes were collected by ultracentrifugation and homogenized in buffer A. The membranes were kept at –80°C until further use.

### Protein Solubilization and Ni-NTA Affinity Chromatography

Stripped membranes were solubilized by a dropwise addition of 280 mM of *N*-nonyl-β-D-glucopyranoside (NG; Anatrace) in buffer A. After overnight incubation at 4°C with gentle agitation, unsolubilized material was sedimented at 40,000 rpm for 30 min at 4°C. To reduce unspecific binding, 20 mM imidazole was added to the solubilized protein and mixed with 1 mL of Ni-NTA agarose slurry (Qiagen) preequilibrated with 20 mM imidazole in buffer B (20 mM HEPES-NaOH pH 8, 500 mM NaCl, 10% Glycerol, 2 mM β-mercaptoethanol and 12 mM NG) and incubated for 16 h with continuous tumbling at 4°C. The resin/protein suspension was loaded onto a poly-prep chromatographic column (Bio-Rad) at 4°C and the flow through containing non-bound protein was removed by gravity flow. The column was washed with buffer B + 70 mM imidazole. The protein was eluted with buffer B + 500 mM imidazole. Since *Nb*XIP1;1α was not stable in the elution buffer after 24 h at 4°C, the eluted protein fraction was desalted and the buffer exchanged for buffer C (20 mM Bis Tris Propane-HCl pH 8.6, 100 mM NaCl, 10% Glycerol, 2 mM β-mercaptoethanol and 12 mM NG) on a PD-10 desalting column (GE Healthcare). The purity of the eluted protein was evaluated by colloidal coomassie staining of SDS-PAGE gels (Neuhoff et al., 1988) and *Nb*XIP1;1α content analyzed by western blot.

### Circular Dichroism (CD) Spectrum

Circular Dichroism spectrum for *Nb*XIP1;1α was acquired as previously published (Plasencia et al., 2011). In brief, the purified *Nb*XIP1;1α or buffer without protein was transferred into a quartz cuvette with a path length of 0.1 cm and the spectra collected at 50 nm/min between 260 and 190 nm with a response time of 8 s and a data pitch of 1 nm in a Jasco Spectrophometer (Jasco, UK) with a built-in Peltier device for sample temperature regulation. Spectrum of the buffer without protein was used for baseline correction.

### Stopped-Flow Spectrometric Assay of Proteoliposomes

To test for boric acid permeability, purified *Nb*XIP1;1α was reconstituted into proteoliposomes by mixing with *Escherichia coli* polar lipid extracts (Avanti Polar lipids) and 20% cholesterol (Sigma) solubilized in 2.5% (w/v) NG in reconstitution buffer (20 mM Tris-HCl pH 8.0, 100 mM NaCl) at a lipid to protein ratio (LPR) of 50 and a final lipid concentration of 2 mg lipids/mL. Approximately 1 g of bio-beads (SM2, Bio-Rad) was added to the lipid/protein solution to remove the detergent. Reconstitution occurred spontaneously as observed by the increase in turbidity of the solution. After 30 min of reconstitution, the supernatant containing the lipid vesicles was collected. The vesicles were extruded nine times through a polycarbonate filter (200 nm pore size) and subjected to an inwardly directed boric acid gradient of 100 mM through rapid mixing in a stopped-flow apparatus. The shrinkage of the vesicles depicting water eﬄux and the subsequent swelling of the vesicles depicting boric acid and water influx was observed by light scattering at 90° angle in a stopped flow apparatus at 500 nm. Control vesicles without protein were subjected to the same steps. One independent reconstitution was performed for each protein and control liposomes were done in parallel. The traces (mean of 8 and 29 traces for the phosphorylated and dephosphorylated protein, respectively) were fitted to double exponential equations. The traces for the control liposomes (mean of 8 and 15 traces for the control liposomes used in the phosphorylated and dephosphorylated reconstitution experiments, respectively) were also fitted to double exponential equations. Kinetic Studio version 2.28 (TgK Scientific Limited) was used to calculate the rate constants and the standard error of estimate (SEE) of the traces.

### Identification of Phosphorylation in NbXIP1;1α with Phos-tag− BTL-111

To probe for phosphorylation in the purified *Nb*XIP1;1α, a phosphate binding tag (Phos-tag− BTL-111, Wako Pure Chemical Industries, Ltd) was utilized as described previously (Kinoshita et al., 2012) with slight modifications. In brief, a complex of Phos-tag− BTL-111 with Horse Radish Peroxidase conjugated Streptavidin (HRP-SA) was prepared as follows; 5 μL of 10 mM aqueous solution of Phos-tag− BTL-111, 20 μL of 10 mM aqueous solution of Zn(NO_3_)_2_, 1 μL of HRP-SA (GE Healthcare Biosciences) and 469 μL of TBS-T (10 mM Tris-HCl pH 7.5, 100 mM NaCl, 0.1% Tween 20) were mixed together and incubated in the dark at ambient temperature. After 1 h of incubation, the mixture was transferred into a 30 K Nanosep filtration column (Pall Corporation) and spun for 10 min at 14,000 ×*g* to remove excess Phos-tag− BTL-111. The remaining solution in the reservoir was diluted with 25 mL of TBS-T solution containing 1% (w/v) Bovine Serum Albumin (BSA) and 1 M sodium acetate and used as a probing reagent (Zn^2+^-Phos-tag− BTL-111-bound HRP-SA solution). Equal amounts of purified *Nb*XIP1;1α untreated and treated with alkaline phosphatase for 20 h at 30°C in 5 mM Tris-HCl (pH 7.9), 10 mM NaCl, 1 mM MgCl_2_, and 0.1 mM DTT, were subjected to SDS-PAGE. The proteins were then transferred onto a PVDF membrane. For probing with the Zn^2+^-Phos-tag− BTL-111-bound HRP-SA, the membrane was blocked with 10% (w/v) BSA in TBS-T solution for 6 h followed by overnight incubation at 4°C with the Zn^2+^-Phos-tag BTL-111-bound HRP-SA solution. The membrane was washed twice for 5 min with TBS-T solution containing 1% (w/v) BSA and 1 M sodium acetate. The blot was developed by enhanced chemiluminescence. Probing with mouse anti-(His)_4_ was done as described above.

### Identification of Phosphorylation Sites in NbXIP1;1α by Mass Spectrometry

To identify phosphorylation sites in the purified *Nb*XIP1;1α, a protein band corresponding to *Nb*XIP1;1 monomeric band was excised from a coomassie-stained SDS-PAGE gel and in-gel digested overnight at 37°C with trypsin (Promega) as published previously (Shevchenko et al., 2006). The trypsin digestion was stopped by the addition of 10% trifluoroacetic acid. The digested peptides were analyzed with an EasyLC nanoflow HPLC interfaced with a nanoEasy spray ion source (Proxeon Biosystems) connected to an LTQ-Orbitrap Velos Pro mass spectrometer (Thermo Fisher Scientific) as described previously (Don-Doncow et al., 2014). The raw data was processed by Mascot distiller search through an in-house Mascot database consisting of amino acid sequences of *N. benthamiana* XIP1;1s, *Arabidopsis thaliana* NIP5;1, and NIP1;1. The parameter settings used in the database search are as follows: fixed modifications: carbidomethyl (C); variable modifications: phospho (ST); enzyme: no enzyme cleavage specificity; maximum missed cleavage sites: one; peptide tolerance: ±6 ppm; MS/MS tolerance: ±0.15 Da. Phosphopeptides identified once and/or with ion score of less than 25 were discarded.

### Phylogenetic Analysis

To construct the alignment, XIP amino acid sequences from *N. tabacum, N. benthamiana, S. tumberosum, S. lycopersicum, I. nil, P. patens*, and *Selaginella moellendorffii* were manually added to a structural alignment of *Bt*AQP1 (1J4N, Sui et al., 2001), *Ec*GLPF (1LDA, Fu et al., 2000), *Ec*AQPZ (1RC2, Savage et al., 2003), *So*PIP2;1 (1Z98, Törnroth-Horsefield et al., 2006), *Rn*AQP4 (2D57, Hiroaki et al., 2006) *Mm*AQPM (2F2B, Lee et al., 2005) *Pf*AQP (3C02, Newby et al., 2008), *Hs*AQP5 (3D9S, Horsefield et al., 2008), and *Bt*AQP0 (2B6P, Gonen et al., 2005) made in DeepView/Swiss-PdbViewer v4.0.1 using MEGA6 (Tamura et al., 2013). The N- and C-termini were aligned with the built-in Muscle algorithm under the default settings (Edgar, 2004).

The phylogenetic tree was inferred using the Maximum Likelihood method based on the Le_Gascuel_2008 model (LG) with discrete Gamma distributions (+G, 5 categories with the parameter 4.8049) and 2.9126% evolutionary invariant sites (+I). All positions with site coverage less than 95% were removed and an initial tree was obtained using the Neighbor-Joining method under a JTT model. The robustness of the nodes was tested by 1000 bootstrap replications.

## Results

### Three XIP Genes in *N. benthamiana*

**FIGURE 1:**
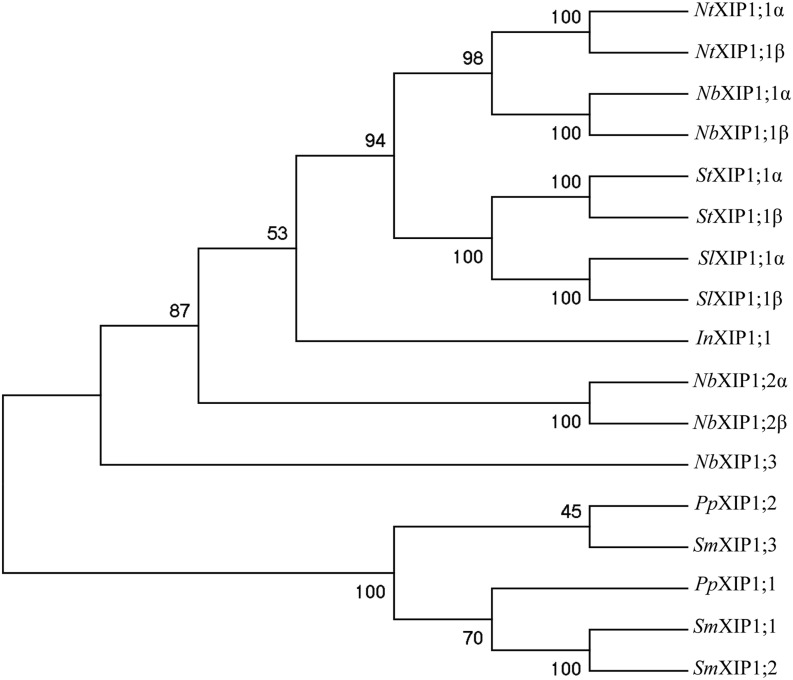
**Phylogenetic tree of XIPs.** The tree shows the phylogenetic relationship between XIPs in *Nicotiana benthamiana*, and selected XIPs of *N. tabaccum*, *S. tuberosum*, *S. lycopersicum*, and *I. nil*, when XIP sequences from *P. patens* and *S. moellendorffii* are used to root the tree. The numbers at the nodes represents the bootstrap support in %. The XIPs of *N. benthamiana, N. tabaccu*m, *S. tumberosum*, *S. lycopersicum*, and *I. nil* represent a subset the XIP IV group of clade B, while *P. patens* and *S. moellendorffii* sequences belong to the basal XIP I group (Venkatesh et al., 2015).

Isoforms belonging to the XIP subfamily of AQPs are present in mosses and in most dicotyledonous plants, while monocotyledonous plants have lost the genes coding for XIP isoforms and so have the dicotyledonous model plant *Arabidopsis thaliana* (Danielson and Johanson, 2008). An examination of the *N. benthamiana* genome revealed three genes coding for full length XIPs, two of which are alternatively spliced. In a phylogenetic analysis the resulting five *N. benthamiana* proteins cluster and form a monophyletic clade together with XIP proteins of other *Solanaceae* species such as *N. tabacum*, *S. tuberosum*, and *S. lycopersicum* (**Figure 1**). This clade also encompass a XIP protein from *I. nil* (Morning glory), whereas XIPs from the moss *P. patens* and the spike moss *S. moellendorffii*, are basal (Danielson and Johanson, 2008; Bienert et al., 2011; Anderberg et al., 2012). Our finding and naming of the *N. benthamiana* XIPs is consistent with what was reported in the course of our work (Venkatesh et al., 2015). In the current study we heterologously expressed the two splice variants α and β of the AQP XIP1;1 from *N. benthamiana*, and subsequently, purified and characterized the α splice-variant.

### Expression of NbXIP1;1 in *Pichia pastoris*

**FIGURE 2:**
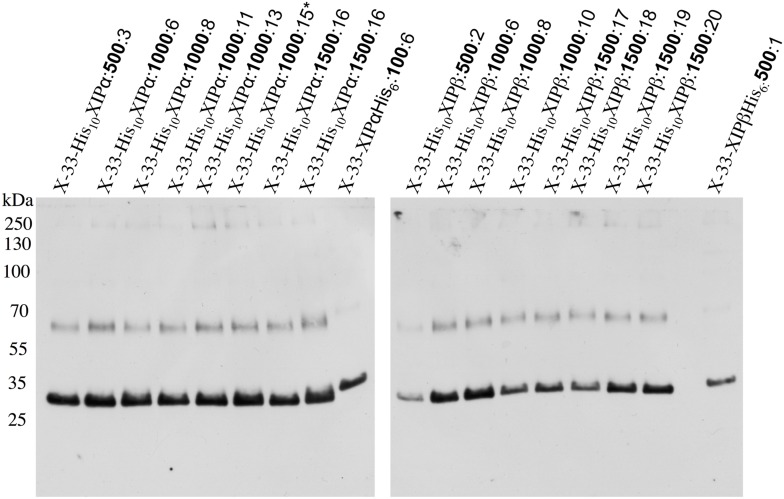
**Recombinant N-terminally His-tagged *Nb*XIP1;1 expression in *P. pastoris* clones selected at different zeocin levels.** Western blot showing the recombinant expression levels in crude cell extracts of representative X-33 N-terminally His-tagged *Nb*XIP1;1 proteins in clones selected at 500 μg/mL, 1000 μg/mL or 1500 μg/mL (μg/mL zeocin resistance levels in bold). C-terminally His-tagged *Nb*XIP1;1 clones were used as reference. The X-33His_10_XIP1;1α:1000:15^∗^ clone was used for large scale purification.

**FIGURE 3:**
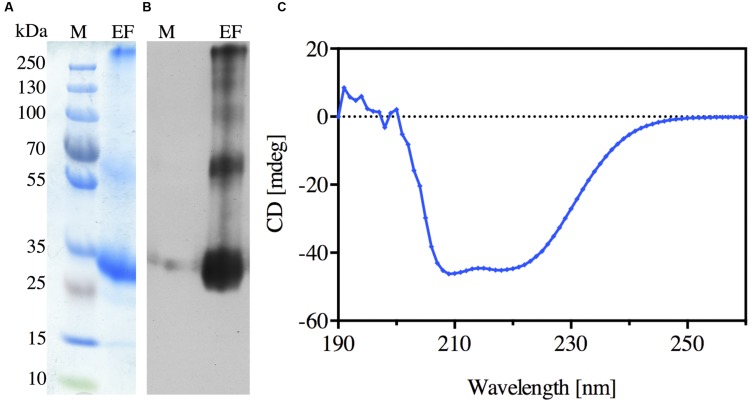
**Purification of the recombinant N-terminally His-tagged *Nb*XIP1;1α protein. (A)** Coomassie stained SDS-PAGE gel showing the elution fraction (EF) from the Ni-NTA Agarose beads. **(B)** Western blot of the elution fraction (EF) showing the purified N-terminally His-tagged *Nb*XIP1;1α protein. **(C)** Far-UV CD spectrum of *Nb*XIP1;1α. The spectrum was measured at 22°C in 20 mM BTP-HCl pH 8.6, 100 mM NaCl, 12 mM *N*-nonyl-β-D-glucopyranoside and 2 mM β-mercaptoethanol.

Both splice variants (*Nb*XIP1;1α and *Nb*XIP1;1β) of *Nb*XIP1;1 were successfully expressed in wild type X-33 (**Figure 2**) and in a protease deficient strain SMD1168H of *P. pastoris* with a slightly higher expression level in the X-33 strain (Supplementary Figure 1). There was no significant difference in the expression levels of the two splice variants. The migration pattern of *Nb*XIP1;1α is typical for AQPs separated by SDS-PAGE (**Figure 3A**; Nordén et al., 2011) with the monomeric and dimeric protein bands running below the expected bands at 37 and 74 kDa, respectively. The selection of multi-copy insertions of the XIP genes were also successful as the clones selected on higher concentrations of zeocin generally had higher protein expression than the clones selected on low concentrations of zeocin (**Figure 2**). The protease deficient SMD1168H clones had a significant reduction in protein degradation (Supplementary Figure 1), however, due to the lower protein expression and yield in the SMD1168H clones as compared to the wild type, the wild-type X-33 *P. pastoris* clones were chosen for further studies. To our knowledge, this is the first time that XIP AQP isoforms have been shown to be successfully expressed in *P. pastoris*.

### *P. pastoris* Cells Expressing NbXIP1;1α Are Sensitive to Boric Acid

**FIGURE 4:**
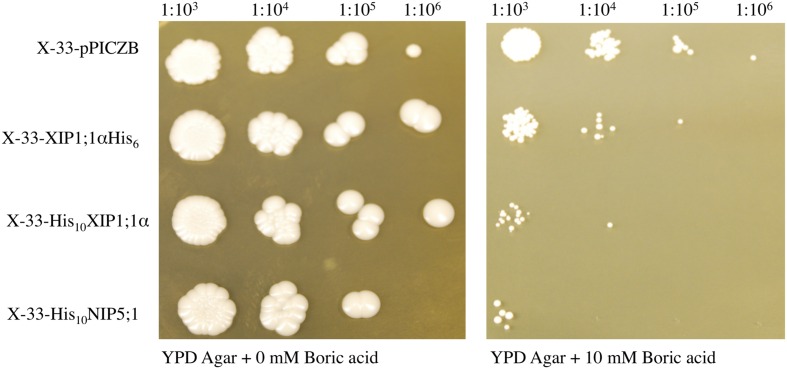
**Boric acid sensitivity of *P. pastoris* cells expressing *Nb*XIP1;1α.** To assess the functionality of the heterologously expressed *Nb*XIP1;1α protein, a growth assay on plates containing 0 mM or 10 mM boric acid was performed. Induced cells, diluted to OD_600_ = 1, were spotted onto the plates in a serial dilution (indicated). X-33 clones transformed with either *At*NIP5;1 or empty pPICZB were included as controls. Growth was recorded after 5 days at 28°C.

*Saccharomyces cerevisiae* yeast mutant (Δ*fps*1) cells expressing a XIP isoform from *N. tabacum* (*Nt*XIP1;1) have previously been shown to possess a growth impeded phenotype when exposed to boric acid (Bienert et al., 2011). Due to the high sequence similarity among the *Nicotiana* XIPs and the relatively close relationship between *S. cerevisiae* and *P. pastoris*, we expected that a boric acid growth assay could be applied to verify the functionality of the heterologously expressed *Nb*XIP1;1α protein. *P. pastoris* cells harboring *Nb*XIP1;1α, *At*NIP5;1 or the empty pPICZB grew to the same extent on the control plates (YPD without boric acid). *At*NIP5;1 was used as a positive control as it has been shown to be a transporter of boric acid (Takano et al., 2006). A *P. pastoris* X-33 clone transformed with empty pPICZB was used as a negative control. The result (**Figure 4**) showed a general reduction in growth of all the cells plated on 10 mM boric acid. However, expression of *Nb*XIP1;1α or *At*NIP5;1 rendered the yeast cells more sensitive to boric acid as there was appreciable inhibition of growth in these clones. This indicated that *Nb*XIP1;1α just like *At*NIP5;1 facilitates the transport of boric acid into the *P. pastoris* cells. As shown in **Figure 4**, switching the His-tag from the C-terminus to the N-terminus of the amino acid sequence appears to increase the sensitivity of the cells to boric acid to a similar level as the positive control *At*NIP5;1.

### NbXIP1;1α Does Not Facilitate Water Transport in *P. pastoris* Spheroplasts

**FIGURE 5:**
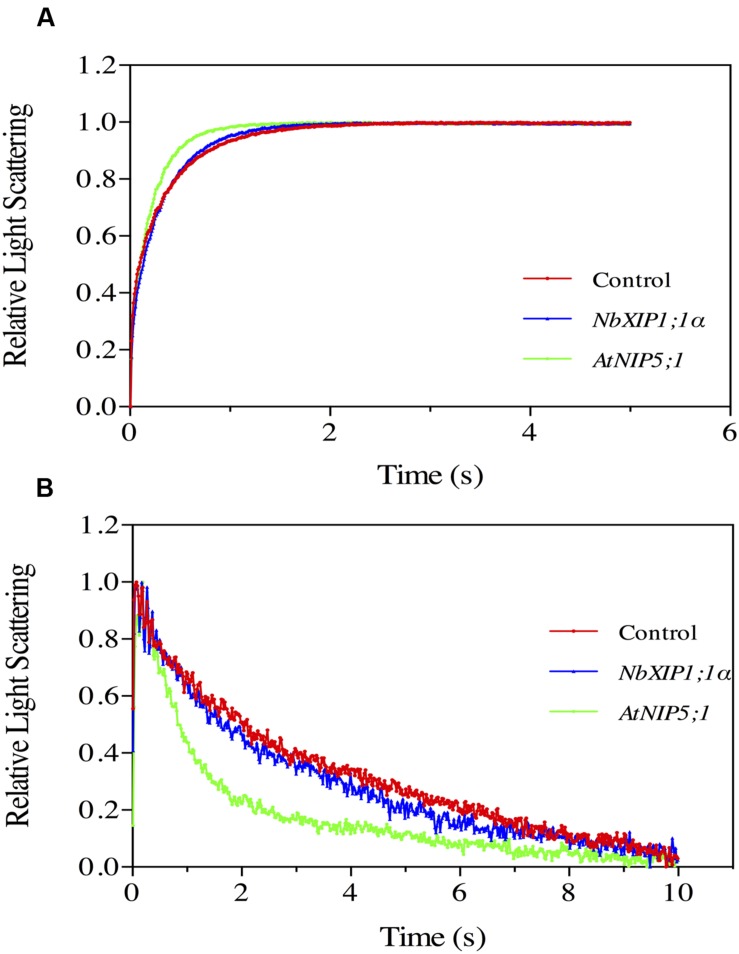
**Substrate specific studies in *P. pastoris* spheroplasts by stopped-flow spectrometry.** Stopped-flow traces showing kinetics of osmotic water permeation **(A)** and glycerol transport **(B)** in control spheroplasts (red), spheroplasts expressing *Nb*XIP1;1α (blue) and spheroplasts expressing *At*NIP5;1 (green). **(A)** The traces (mean of at least 10 traces) were fitted to single exponential equations. The rate constant and the standard error of estimate (SEE) values for the fitted curves were 2.77 ± 0.04 s^–1^ (control); 3.20 ± 0.03 s^–1^ (*Nb*XIP1;1α) and 4.90 ± 0.04 s^–1^ (*At*NIP5;1). **(B)** The result shows a fast increase in light scattering followed by a gradual decrease in light scattering corresponding to a fast vesicle shrinkage due to water eﬄux and a gradual vesicle swelling due to glycerol and water influx, respectively. The rate constant and SEE values for the fitted curves after fitting the traces after 0.39 s (mean of at least seven traces) to single exponential equations were 0.213 ± 0.004 s^–1^ (control); 0.301 ± 0.005 s^–1^ (*Nb*XIP1;1α) and 0.742 ± 0.015 s^–1^ (*At*NIP5;1).

The ability of *Nb*XIP1;1α to facilitate the transport of water was tested by subjecting *P. pastoris* spheroplasts heterologously expressing *Nb*XIP1;1α to a stopped-flow spectrometric analysis. After fitting the data to a single exponential function, the rate constants ± standard error of estimate (SEE) for water permeation through the control transformed with the empty pPICZB plasmid, *Nb*XIP1;1α and *At*NIP5;1 spheroplasts were calculated as: 2.77 ± 0.04 s^–1^; 3.20 ± 0.03 s^–1^; and 4.90 ± 0.04 s^–1^, respectively. This suggested that there was, if any, little or no difference in water permeability between the *Nb*XIP1;1α and control spheroplasts as shown in **Figure 5A**. However, the *At*NIP5;1 spheroplasts had an approximately twofold increase in water permeation as compared to the control spheroplasts.

### NbXIP1;1α Does Not Facilitate Glycerol Transport in *P. pastoris* Spheroplasts

In previous studies, *Nt*XIP1;1 was shown to facilitate the transport of glycerol in *Xenopus oocytes* (Bienert et al., 2011). In the light of that, glycerol transport by the recombinant *Nb*XIP1;1α in *P. pastoris* spheroplasts was assayed by stopped-flow spectrometry (**Figure 5B**). The result shows a fast increase in light scattering followed by a gradual decrease in light scattering corresponding to a fast vesicle shrinkage due to water eﬄux and a gradual vesicle swelling due to glycerol and water influx, respectively. The rate constants for glycerol permeation through the control spheroplasts, spheroplasts expressing *Nb*XIP1;1α and *At*NIP5;1 were calculated as: 0.213 ± 0.004 s^–1^; 0.301 ± 0.005 s^–1^; and 0.742 ± 0.015 s^–1^, respectively. The result indicated that there was little or no substantial difference in glycerol permeability between the *Nb*XIP1;1α and the control spheroplasts as shown in **Figure 5B**. However, the *At*NIP5;1 had approximately a 3.5-fold increase in glycerol permeation as compared to the control spheroplasts.

### Plasma Membrane Localization of NbXIP1;1α in *P. pastoris* Cells

**FIGURE 6:**
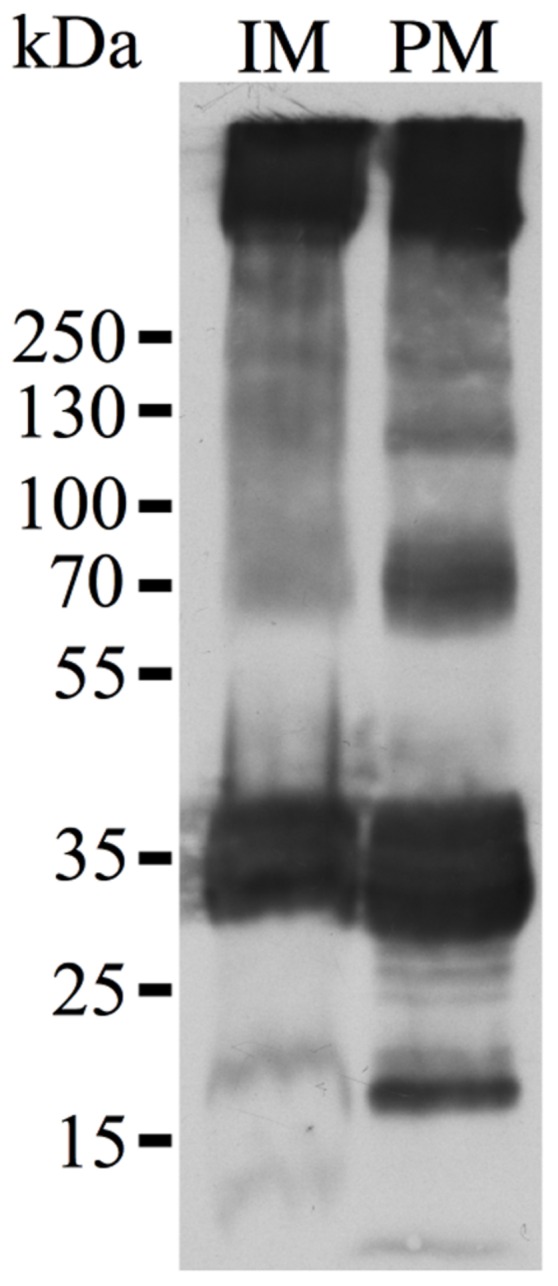
**Plasma membrane localization of *Nb*XIP1;1α in *P. pastoris*.** Western blot analysis of membrane fractions of *P. pastoris* cells expressing the N-terminally His-tagged *Nb*XIP1;1α protein. In accordance with previous results using the anti-(His)_4_ antibody (Nordén et al., 2011), the blot shows a pattern that is characteristic of the expressed protein. IM, internal membranes fraction. PM, plasma membrane fraction. The fractions were diluted to the same volume and an equal volume was loaded in the two lanes.

XIP isoforms were predicted (Danielson and Johanson, 2008) and later confirmed to be localized in the plasma membrane of plant cells (Bienert et al., 2011). However, to discard the likelihood that the inability to transport water and glycerol in the *Pichia* spheroplasts was due to lack of expression or to a disturbance in the trafficking to the plasma membrane in the yeast, the localization of the recombinant N-terminally His_10_-tagged *Nb*XIP1;1α in *P. pastoris* X-33 cells was investigated. Sucrose gradient fractions of cell homogenates of *P. pastoris* cells expressing *Nb*XIP1;1α analyzed by western blots indicated that *Nb*XIP1;1α was present not only in the internal membranes (including the ER) fraction but also in the plasma membrane fraction as shown in **Figure 6**. This further indicated that the trafficking of the recombinant *Nb*XIP1;1α from the ER to the plasma membrane was not impeded in the *P. pastoris* cells.

### Purification and CD Spectrum of Recombinant NbXIP1;1α

*N*-nonyl-β-D-glucopyranoside detergent was used to solubilize *Nb*XIP1;1α from urea stripped *P. pastoris* membranes. The solubilized protein was purified further by affinity chromatography, utilizing the His-tag at the N-terminus of the recombinant *Nb*XIP1;1α. The elution fraction from the chromatography proved to be highly enriched in *Nb*XIP1;1α protein as shown in **Figures 3A,B**. Using the current optimized protocol, a yield of 0.15 mg protein per gram of yeast cells was routinely obtained.

A CD spectrum of the purified *Nb*XIP1;1α protein was recorded to verify that it was intact and correctly folded. CD spectra of AQPs mainly identify the α-helical content of the proteins since AQPs are composed of six α-helices that traverse the membrane with interconnecting loops (Plasencia et al., 2011). As shown in **Figure 3C**, the characteristic CD pattern at 209 and 222 nm indicated that the purified *Nb*XIP1;1α protein was mainly α-helical and correctly folded, and may therefore be reconstituted into artificial proteoliposomes for substrate specificity studies.

### Boric Acid Permeation in Proteoliposomes

**FIGURE 7:**
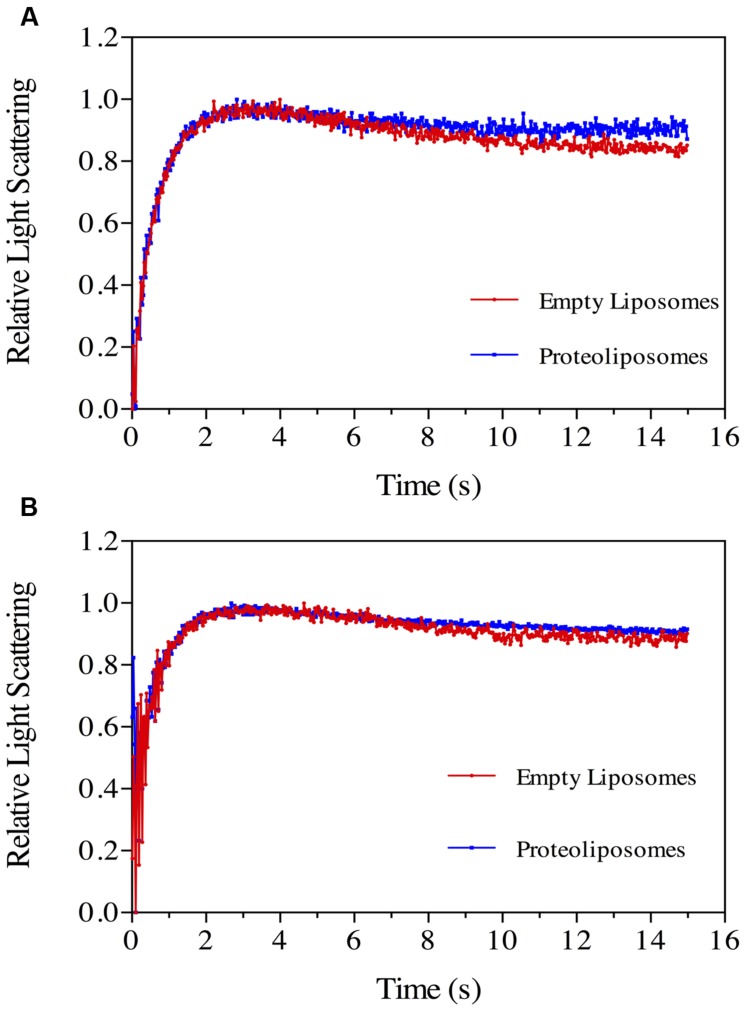
**Boric acid permeability in proteoliposomes.** Stopped-flow traces showing kinetics of boric acid transport in phosphorylated **(A)** and dephosphorylated **(B)** *Nb*XIP1;1α protein reconstituted in *E. coli* lipid vesicles supplemented with 20% cholesterol. Control liposomes (red) and proteoliposomes (blue). **(A)** The traces (mean of eight traces) were fitted to double exponential equations. The rate constant and SEE values for the fitted curves were control (R_1_ = 1.47 ± 0.03 s^–1^; R_2_ = 0.11 ± 0.01 s^–1^) and *Nb*XIP1;1α (R_1_ = 1.62 ± 0.05 s^–1^; R_2_ = 0.20 ± 0.03 s^–1^). **(B)** The rate constant and SEE values for the fitted curves after fitting the traces (mean of 15 and 29 traces for control liposomes and proteoliposomes, respectively) to double exponential equations were control (R_1_ = 1.44 ± 0.07 s^–1^; R_2_ = 0.11 ± 0.04 s^–1^) and *Nb*XIP1;1α (R_1_ = 1.02 ± 0.10 s^–1^; R_2_ = 0.26 ± 0.07 s^–1^).

Boric acid permeation through the *Nb*XIP1;1α protein reconstituted into *E. coli* polar lipid vesicles supplemented with 20% cholesterol was monitored by stopped-flow spectrometry. An initial inwardly directed boric acid gradient of 100 mM resulted in an initial water eﬄux followed by boric acid and water influx. This resulted in an initial shrinkage followed by swelling of the vesicles as shown in **Figure 7A**. After fitting the data to a double exponential function, the second rate constants (which estimate the rate at which boric acid permeate through the vesicles) of the control vesicles and proteoliposomes were calculated as 0.11 ± 0.01 s^–1^ and 0.20 ± 0.03 s^–1^, respectively. This indicated that the purified *Nb*XIP1;1α was not fully active as it could only manage a twofold increase in boric acid permeation through the vesicles.

### Recombinant NbXIP1;1α Is Phosphorylated in *P. pastoris*

**FIGURE 8:**
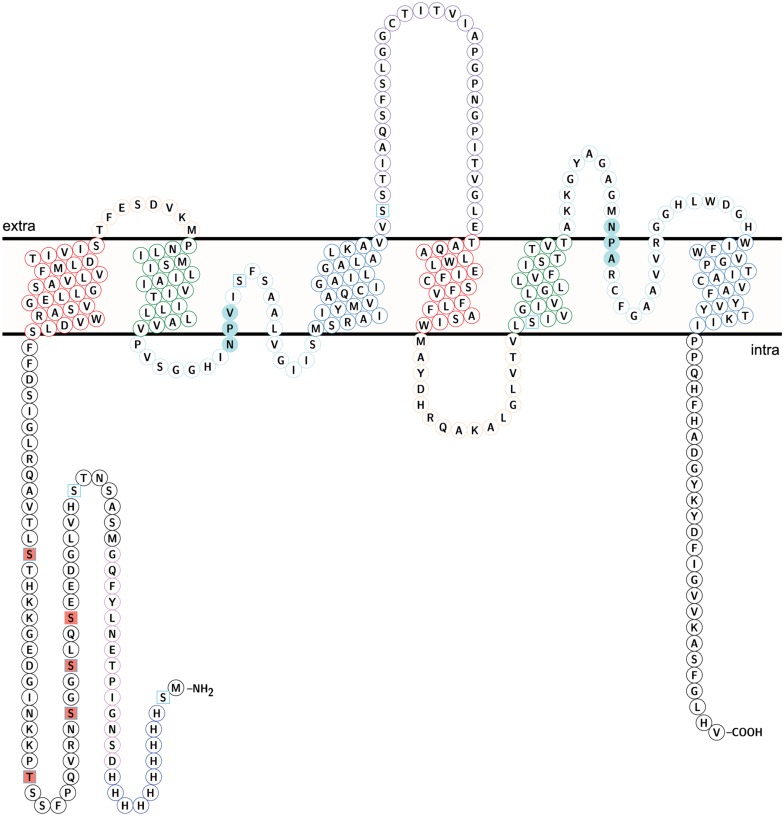
**Topology of *Nb*XIP1;1α protein showing identified phosphorylation sites.** Identified phospho sites – square salmon fill. Predicted phospho sites – square frame in sky blue. The coloring of helices and loops reflect a direct repeat in the gene/protein. Transmembrane helix (TM) 1 and TM 4 – frame in red. TM2 and TM 5 – frame in green. TM 3 and TM 6 – frame in steel blue. Loop A and Loop D – frame in peach. Loop B and loop E – frame in pale turquoise. Loop C – frame in medium purple. NPV/NPA box – pale turquoise fill. H3-H12 – Histidine tag in blue. D13-G26 – TEV protease cleavage site and linker in violet. The topology model was done in Protter (Omasits et al., 2014).

**FIGURE 9:**
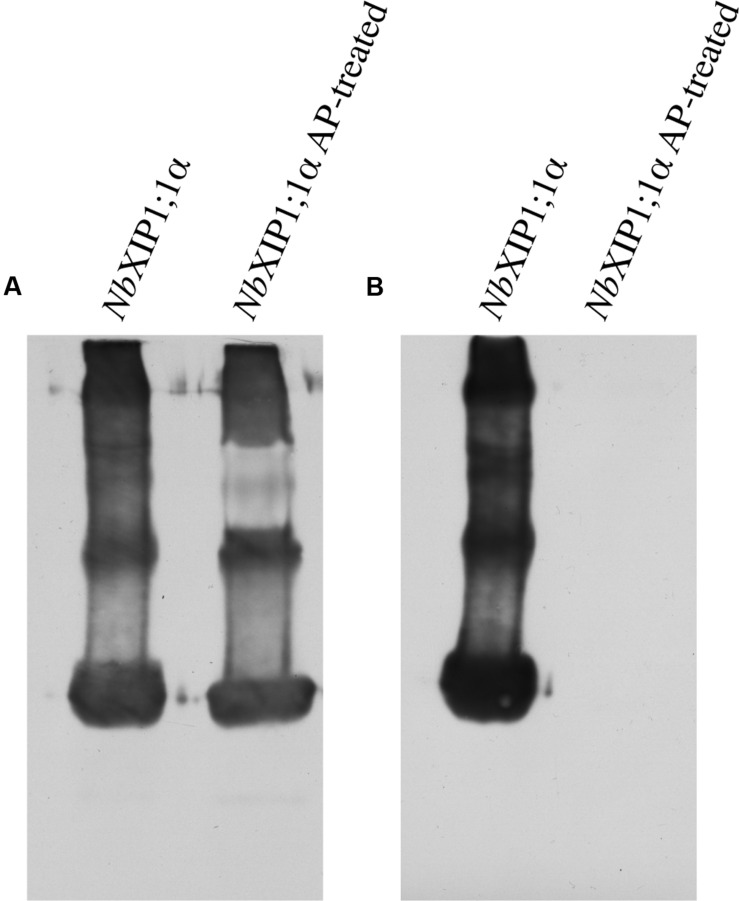
**Phosphorylation in purified *Nb*XIP1;1α protein. (A)** Western blot showing purified *Nb*XIP1;1α and purified *Nb*XIP1;1α treated with alkaline phosphatase (AP) in 5 mM Tris-HCl, pH 7.9, 10 mM NaCl, 1 M MgCl_2_ and 0.1 mM DTT at 30°C for 20 h. Blot was probed with mouse anti-(His)_4_ and Horse Radish Peroxidase (HRP) – conjugated polyclonal goat anti-mouse IgG as primary and secondary antibodies, respectively. **(B)** Western blot showing purified *Nb*XIP1;1α and purified *Nb*XIP1;1α treated with AP as in **(A)** and probed with Zn^2+^-Phostag BTL-111-bound HRP-Streptavidin.

Attempts to render the recombinant *Nb*XIP1;1α protein fully active led to the investigation of the phosphorylation status of the purified protein, since phosphorylation has previously been shown to regulate AQP function (Kuwahara et al., 1995; Maurel et al., 1995; Johansson et al., 1998). Interestingly, an *in silico* phosphorylation prediction tool NetPhosYeast (Ingrell et al., 2007) revealed that the N-terminal part of *Nb*XIP1;1α harbors six putative phosphorylation sites as shown in **Figure 8**. Probing for phosphorylation with the Phostag− BTL-111 confirmed that indeed the purified *Nb*XIP1;1α protein was phosphorylated as shown in **Figure 9B**. It is also important to note in **Figure 9A** that alkaline phosphatase dephosphorylated the purified *Nb*XIP1;1α, making the protein bands appear sharper and more homogenous. Dephosphorylation of the purified *Nb*XIP1;1α by alkaline phosphatase had little or no effect on the functionality of the protein as there was no appreciable change in boric acid transport when reconstituted into *E. coli* lipid vesicles as shown by the second rate constants (untreated 0.20 ± 0.03 s^–1^, dephosphorylated 0.26 ± 0.07 s^–1^; **Figures 7A,B**). A direct interpretation would be that the partially phosphorylated protein is only partly open, or that only a subfraction of *Nb*XIP1;1α is sufficiently phosphorylated to be open.

### Phosphorylation Sites in NbXIP1;1α Protein

To identify the sites of phosphorylation in *Nb*XIP1;1α, mass spectrometry (MS) was applied. A sequence coverage of 65% was obtained for *Nb*XIP1;1α in the MS analysis as shown in Supplementary Figure 2. Detected peptides are also shown in Supplementary Table 1. All the five phosphorylation sites [four S(p) and one T(p)] identified were in the N-terminal region of the *Nb*XIP1;1α protein (**Figure 8** and **Table 1**) and four of these coincided with the nine predicted phosphorylation sites. The MS data showed that the purified recombinant *Nb*XIP1;1α protein was partially phosphorylated as identical peptides were found with or without phosphorylation (Supplementary Figure 3).

**Table 1:** Phosphorylation sites in *Nb*XIP1;1α identified by mass spectrometry.

Number	Assigned p-sites	Sequence	Mascot Score	Charge	Experimental weight	Theoretical weight
26–49	S41	GMSASNTSHVLGDEES(p)QLSGGSNR	95	3+	2499.0246	2499.0333
28–49	S44	SASNTSHVLGDEESQLS(p)GGSNR	92	3+	2310.9612	2310.9714
32–49	S47	TSHVLGDEESQLSGGS(p)NR	82	2+	1951.8163	1951.8273
50–58	T56	VQPFSST(p)PK	42	2+	1069.4796	1069.4845
68–76	S70	HTS(p)LTVAQR	63	2+	1091.5076	1091.5125

## Discussion

### Heterologous Expression

**FIGURE 10:**
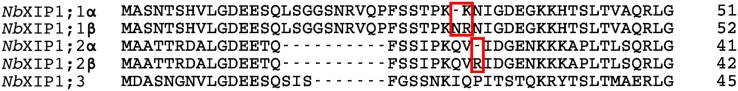
**Close up of the splice sites in the amino acid sequences of *Nb*XIP1 splice variants.** Splice sites in *Nb*XIP1;1 splice variants and *Nb*XIP1;2 splice variants are in red boxes.

Just as for the *N. tabacum* XIP1;1 splice variants (Bienert et al., 2011), the difference between the two *N. benthamiana* splice variants is that the *Nb*XIP1;1β splice-variant is one amino acid longer as compared to the *Nb*XIP1;1α splice-variant. This is due to the alternative splice site in the first intron either comprised of the donor and acceptor sites GT … AG for *Nb*XIP1;1β or GT … (AGC) AG for *Nb*XIP1;1α (Supplementary Figure 4), resulting in the *Nb*XIP1;1β splice-variant being one arginine longer. The shorter splice-variant *Nb*XIP1;1α looses the arginine and the amino acid upstream in this splice-variant changes from an asparagine to a lysine, with all other amino acids left identical in the two splice variants (**Figure 10**).

In order to generate enough protein for structural and functional characterizations of the *Nb*XIP1;1 proteins, cDNAs corresponding to both splice variants were transformed into the yeast *P. pastoris*. After optimizing the protein expression levels, by screening clones with different gene copy numbers selected at different levels of antibiotic concentration (Nordén et al., 2011), both splice variants expressed at similar protein levels. Not all AQPs express well in *P. pastoris* but for these two isoforms the expression levels were relatively high and comparable to other plant and human AQPs and to other integral membrane proteins that we have managed to express and purify in functional form (Karlsson et al., 2003; Törnroth-Horsefield et al., 2006; Horsefield et al., 2008; Agemark et al., 2012; Moparthi et al., 2014; Kirscht et al., 2016). Due to a slightly higher expression level, the shorter splice-variant *Nb*XIP1;1α was selected for further investigations in the present study (Supplementary Figure 1).

In most plant organs and tissues XIP isoforms have been predicted and shown to be located in the plasma membrane (Danielson and Johanson, 2008; Bienert et al., 2011). In order to functionally study *Nb*XIP1;1α in the cell membrane of *P. pastoris* or purified from a crude membrane fraction from *P. pastoris*, it is necessary to verify that this AQP is correctly targeted to the *P. pastoris* cell membrane. When membrane vesicles from a total membrane fraction (i.e., a microsomal fraction) of *P. pastoris* were separated on a sucrose density gradient, *Nb*XIP1;1α was shown to be primarily located in the plasma membrane fraction (**Figure 6**). As expected, the protein was also to a lesser extent present in the internal membrane fraction since *Nb*XIP1;1α, like all integral proteins of the plasma membrane, is synthesized in the ER.

### Substrate Selectivity of NbXIP1;1α in *P. pastoris*

The orthodox AQPs are only permeable to water, e.g., *Hs*AQP1 and *So*PIP2;1, (Preston et al., 1992; Törnroth-Horsefield et al., 2006), while other AQPs are permeable to water and in addition other small uncharged solutes, e.g., glycerol, (*Ec*GlpF; Fu et al., 2000), ammonia (*At*TIP2;1; Kirscht et al., 2016), and boric acid (*At*NIP5;1; Takano et al., 2006). The substrate specificity of AQPs is determined by a selectivity filter that consists of two regions in the pore of AQPs.

One is the NPA region that basically prevents protons from going through the pore. The NPA region is formed by two half helices each of which have the amino acids NPA at their N-termini. The N-termini of the two half helices meet and connect in the middle of the membrane spanning AQP creating a partial positive charge in the pore that prevents protons from passing. Based on sequence alignments the XIPs conform to the generic AQP fold with the two half helices and for most XIPs, including the *Nb*XIP1;1s, the NPA boxes are relatively well conserved. It is only in the third position of the first NPA box that the alanine is substituted for a valine (Supplementary Figure 5) or another small uncharged amino acid residue in other plant species (Danielson and Johanson, 2008). Thus it is likely that there is an obstruction for hydronium ion permeation also in the XIPs. This is consistent with our finding that *Nb*XIP1;1α is tolerated in the plasma membrane where a leakage of protons would be detrimental for the pH and charge gradients that are crucial for nutrient uptake and maintenance of the osmotic balance.

The other region in the pore that determines the substrate specificity of AQPs is the so-called aromatic/arginine (ar/R) region. This region has recently been extended to consist of five amino acids at specific positions in the pore and the identity of these amino acids determine which substrates can pass (**Table 2**; Kirscht et al., 2016). It should be noted that the sequence divergence of XIPs from other AQPs makes the identity of the amino acid residues at position LC^P^ in C-loop and H5^P^ in helix 5 highly uncertain, and it is likely that a structure of a XIP will reveal novel and unpredicted features, as the structure of *At*TIP2;1 did for the TIP subfamily. The amino acids predicted to constitute the ar/R region of *Nb*XIP1;1α are similar to the amino acids in the ar/R region of some of the AQP isoforms belonging to the NIP subfamily in plants. One of these isoforms is *At*NIP5;1 that has been shown to be permeable to boric acid, as mentioned above (Takano et al., 2006).

**Table 2:** Aromatic arginine (ar/R) selectivity filter of *Nb*XIP isoforms, human and *A. thaliana* aquaporin isoforms.

AQPs	H2^P^	LC^P^	H5^P^	LE^P^	HE^P^
*Nb*XIP1;1s	I	C	V/T	A	R
*Nb*XIP1;2s	A	C	V/T	A	R
*Nb*XIP1;3	I	C	V/T	A	R
*At*NIP1s, 2;1, 4s	W	S/T	V	A	R
*At*NIP3;1	W	T	I	A	R
*At*NIP5;1	A	T	I	G	R
*At*NIP6;1	A	T	I	A	R
*At*NIP7;1	A	T	V	G	R
*At*TIP1s	H	F	I	A	V
*At*TIP2s	H	H	I	G	R
*At*TIP3s	H	F	I	A	R
*At*TIP5;1	N	Y	V	G	C
*Hs*AQP8	H	F	I	G	R
*At*PIPs	F	N	H	T	R
*At*SIP1;1	I	G	V	P	I
*At*SIP1;2	V	G	F	P	I
*At*SIP2;1	S	K	H	G	A

*The ar/R filter is defined by five amino acid residues: one in helix 2 (H2^P^), one in loop C (LC^P^), one in helix 5 (H5^P^), one in loop E (LE^P^), and one in helix E (HE^P^). ^P^The position of the amino acid residue in the ar/R filter. Due to alternative alignment possibilities, the amino acids in the LC^P^ and H5^P^ positions are uncertain.*

### Boric Acid

We used a growth assay in *P. pastoris* to investigate whether also *Nb*XIP1;1α is permeable to boric acid (B[OH]_3_) and found that the cells expressing the *Nb*XIP1;1α AQP were similarly sensitive to boric acid as compared to the cells expressing the *At*NIP5;1. Thus, *Nb*XIP1;1α is permeable to boric acid and placing the His-tag at the N-terminal renders the protein slightly more permeable to boric acid as compared to a channel with a C-terminally located His-tag (**Figure 4**). This result may suggest that the N-terminally His-tagged *Nb*XIP1;1α is somewhat more open, which might have implications when discussing a putative gating mechanism involving phosphorylation of the protein (see below). However, the difference in sensitivity of the cells may be due to difference in the expression levels of *Nb*XIP1;1α constructs as the N-terminally His-tagged protein expresses better than the C-terminally His-tagged protein as shown in **Figure 2**. That *Nb*XIP1;1α in the growth assay is equally permeable to boric acid as compared to *At*NIP5;1 also suggests that *Nb*XIP1;1α is not only correctly targeted to the cell membrane but also correctly folded and functional.

### Water and Glycerol

Whether *Nb*XIP1;1α is permeable to water is possible to investigate using *P. pastoris* spheroplasts and stopped-flow spectrometry. The results suggest that water flow through *Nb*XIP1;1α do not take place or is very limited (**Figure 5A**), while the control boric acid-permeable AQP *At*NIP5;1 displayed a comparably higher but limited water permeability. However, the sizes of the different spheroplasts were not checked and this may complicate the interpretation of the result. In a previous study, *Pt*XIP2;1 and *Pt*XIP3;3 were permeable to water when expressed in *Xenopus oocytes* whereas other *Pt*XIPs including *Pt*XIP1;1 were not permeable to water (Lopez et al., 2012).

Some XIP isoforms have been reported to be permeable to glycerol when transiently expressed in *Xenopus laevis* oocytes (Bienert et al., 2011). Using the *P. pastoris* spheroplast experimental system, we could not detect any glycerol permeability for *Nb*XIP1;1α while the control AQP *At*NIP5;1 showed approximately a fourfold higher glycerol permeability as compared to spheroplasts from *P. pastoris* cells transformed with empty pPICZB plasmid (**Figure 5B**). Our spheroplast results for *Nb*XIP1;1α are in agreement with previous results showing that XIP1;1 isoforms are not permeable to water when expressed in oocytes (Bienert et al., 2011; Lopez et al., 2012). However, the glycerol permeability that could be seen for XIP1;1 isoforms when expressed in oocytes was not evident using the *P. pastoris* spheroplast experimental system. Whether this discrepancy reflects the choice of the different experimental systems or a difference in the substrate specificity of the different XIP1;1 isoforms from *N. benthamiana, N. tabacum*, and *P. trichocarpa*, respectively, remains unclear.

It is quite difficult to visualize how small uncharged substances like boric acid can permeate through an AQP pore without water also being transported. Nevertheless, the inability of *Nb*XIP1;1α to facilitate the transport of water was not unexpected considering the amino acids defining the selectivity filter, since the amino acid residues constituting its ar/R region are more hydrophobic than the amino acids constituting the ar/R region of orthodox water permeable AQPs (Danielson and Johanson, 2008; Bienert et al., 2011). Based on the above, we suggest that it is unlikely that water is a true *in vivo* substrate for XIP1;1 isoforms and furthermore, that it is still unclear whether glycerol is a true *in vivo* substrate, especially since the relevance for glycerol transport in plants is limited. This is in contrast to mammalian cells were glycerol from hydrolysed triacylglycerols need to be transported and taken up by liver cells and converted to glucose (Jelen et al., 2011). One instance when triacylglycerols are hydrolysed in plants is during seed germination releasing fatty acids that by β-oxidation provide energy for germination. Another plant developmental state when fatty acids from triacylglycerols provide energy is during pollen tube growth following pollination (Buchanan et al., 2000). Upon triacylglycerol hydrolysis in seeds, the fatty acids and the remaining glycerol are released from the oil bodies into the cytosol. The glycerol may be converted to triose phosphates and subsequently to sucrose, but this takes place inside the cells. Thus, it remains unclear whether glycerol has to be transported between cells and require glycerol-specific protein channels in the plasma membrane of cells.

### Characterization of Purified NbXIP1;1α

The N-terminally His-tagged *Nb*XIP1;1α solubilized from *Pichia* membranes and Ni-NTA affinity purified, showed a typical AQP banding pattern when separated by SDS-PAGE with the monomeric form as the dominating band (**Figures 3A,B**). The CD spectrum of *Nb*XIP1;1α was very similar to that of other AQPs showing a typical CD spectrum of a predominantly α-helical protein, suggesting that the α-helices are intact and that *Nb*XIP1;1α is correctly folded (**Figure 3C**). The purified *Nb*XIP1;1α was reconstituted into proteoliposomes in order to be able to monitor the function of the isolated protein, i.e., XIP1;1α homotetramers, in contrast to the *P. pastoris* spheroplast experimental set up in which putatively interfering proteins might be present. The stopped-flow spectrometry measurements showed that *Nb*XIP1;1α conferred a twofold increase in the boric acid permeation as compared to empty liposomes (**Figure 7A**). This result is in contrast to the apparently highly active boric acid transport by *Nb*XIP1;1α in the *P. pastoris* cells. This suggests to us that even if *Nb*XIP1;1α is permeable to boric acid when reconstituted into proteoliposomes, the *Nb*XIP1;1α pore is most probably not fully open. This could be due to a yet unknown gating mechanism blocking the pore, such that only a subpopulation of the *Nb*XIP1;1α present in the proteoliposomes have an open pore and that the rest have a closed conformation. The absence of an accessory protein necessary for the pore to be fully open *in vivo* would also result in a low channel activity.

### Phosphorylation of NbXIP1;1α

It is known from other plant AQPs that phosphorylation and dephosphorylation of specific amino acids, such as serines and threonines, is involved in opening and closing the pore (Johansson et al., 1998; Törnroth-Horsefield et al., 2006). In order to discern whether the difference in boric acid permeability of *Nb*XIP1;1α when present in *P. pastoris* cells as compared to reconstituted into proteoliposomes could be due to different phosphorylation states of the protein, we first investigated whether purified *Nb*XIP1;1α was phosphorylated. **Figure 9** clearly shows that purified *Nb*XIP1;1α is indeed phosphorylated and that the phosphate groups can be removed by alkaline phosphatase. To define which specific amino acids are phosphorylated, we used an *in silico* prediction tool in combination with mass spectrometry. The protein was predicted to be phosphorylated at nine different serines and threonines, six in the N-terminal region, one serine in loop B, one serine in loop C and one serine in helix 5 (**Figure 8**). By mass spectrometry four of these predicted phosphorylation sites were confirmed, all four in the N-terminal region. In addition, the mass spectrometry analysis discerned yet another phosphorylation site, also in the N-terminal region. Thus, *Nb*XIP1;1α is phosphorylated at altogether five amino acids in the N-terminal region, four serines and one threonine. Since phosphorylation of AQPs has been shown to be important for both gating the channel, as mentioned above, as well as for targeting the AQP to the plasma membrane, phosphorylation of some of the amino acids might have a role in targeting *Nb*XIP1;1α to the plasma membrane. Interestingly, the N-terminus of *At*NIP5;1, an AQP isoform that shares similar substrate specificity with the *Nt*XIPs, needed to be truncated to be active in *S. cerevisiae* yeast cells (Bienert et al., 2008). Here, we show that a full-length version is successfully expressed and active in the plasma membrane of *P. pastoris*. This deviance may be due to the presence of the added N-terminal His-tag or due to differences in trafficking mechanisms in the two yeasts, possibly directed by phosphorylation.

Both phosphorylated and non-phosphorylated, but otherwise identical, *Nb*XIP1;1α peptides were identified by mass spectrometry (Supplementary Figure 3). This suggests that only a fraction of *Nb*XIP1;1α was phosphorylated. When a dephosphorylated *Nb*XIP1;1α was reconstituted into proteoliposomes, no difference in boric acid permeability could be detected compared to phosphorylated *Nb*XIP1;1α (**Figure 7B**). We speculate that the dephosphorylated *Nb*XIP1;1α represents the closed conformation of the protein and the N-terminal region may need to be fully phosphorylated for the channel to open. For other AQPs, it has been shown that amino acids phosphorylated when the proteins were expressed in *P. pastoris* were also phosphorylated *in planta* (Daniels and Yeager, 2005). Thus, the amino acids in the N-terminal region of *Nb*XIP1;1α shown here to be phosphorylated by kinases in *P. pastoris* would most likely also be accessible for plant kinases. Whether *Nb*XIP1;1 is phosphorylated *in planta* and if this is affected by the alternative splicing, remains to be shown but it could potentially be a mechanism that regulates trafficking or gating of *Nb*XIP1;1.

## Conclusion

We have shown that XIP1;1 isoforms can be expressed in the heterologous host *P. pastoris*, that the splice variant *Nb*XIP1;1α is functional and permeable to boric acid in *Pichia* cells and that *Nb*XIP1;1α is not a water channel. Moreover, we have shown that the N-terminal region of *Nb*XIP1;1α is phosphorylated. We suggest that *Nb*XIP1;1α is gated by phosphorylation and that the phosphorylated protein represents the open conformation.

## Author Contributions

PK and UJ conceived the research. PK and UJ planned the experiments. HA-K, HA, AE, AK, KN, and SK performed the experiments. HA-K wrote the manuscript. PK, UJ, HA-K, KN, and SK revised the manuscript.

## Conflict of Interest Statement

The authors declare that the research was conducted in the absence of any commercial or financial relationships that could be construed as a potential conflict of interest.
