# Supplementary material for: The Aquaporin Splice Variant NbXIP1;1α Is Permeable to Boric Acid and Is Phosphorylated in the N-terminal Domain
Source: Front Plant Sci. 2016 Jun 16;7:862. doi: 10.3389/fpls.2016.00862 (PMC4909777; doi:10.3389/fpls.2016.00862)
Supplement: Supplementary file 1 [file Presentation_1.PDF]

Supplementary Material

The aquaporin splice variant *Nb*XIP1;1α is permeable to boric acid and is phosphorylated in the N-terminal domain

Henry Ampah-Korsah[1], Hanna I. Anderberg[1], Angelica Engfors[1], Andreas Kirscht[1], Kristina Nordén[1], Sven Kjellström[1], Per Kjellbom[1] & Urban Johanson[1]*

*** Correspondence:** Urban.Johanson@biochemistry.lu.se

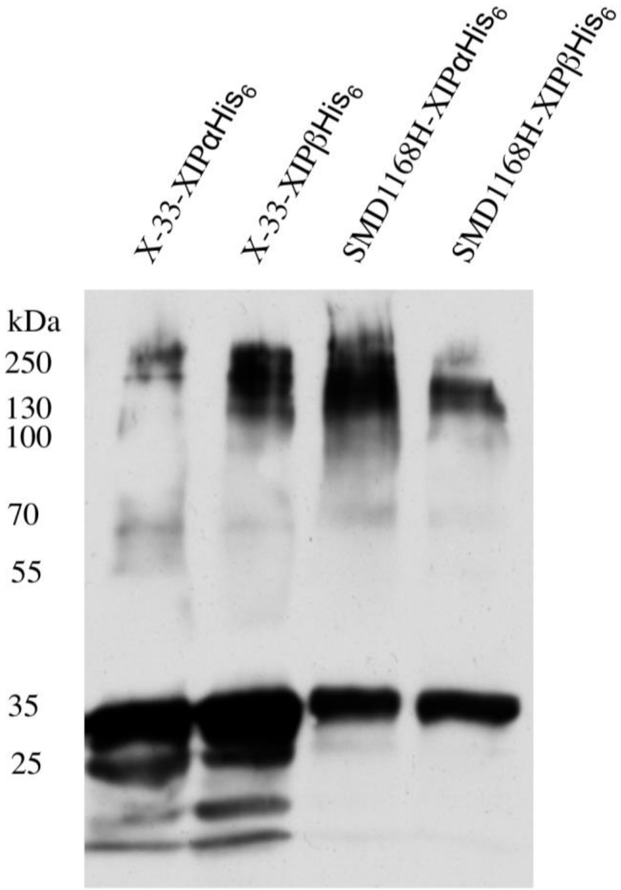

Supplementary Figure 1. Expression in protease deficient SMD1168H cells to reduce degradation. Western blot showing expression level and degradation degree of the two *Nb*XIP1;1 splice versions in crude cell extracts of wild-type X-33 and protease deficient SMD1168H *P. pastoris* cells.

Protein sequence coverage: **65%**

Matched peptides shown in **Bold Red**

```
  1 MSHHHHHHHH HHDSNGIPTE NLYFQGMSAS NTSHVLGDEE SQLSGGSNRV
 51 QPFSSTPKKN IGDEGKKHTS LTVAQRLGIS DFFSLDVWRA SVGELLGSAV
101 LVFMLDTIVI STFESDVKMP NLIMSILIAI VITILLLAVV PVSGGHINPV
151 ISFSAALVGI ISMSRAIIYM VAQCIGAILG ALALKAVVSS TIAQSFSLGG
201 CTITVIAPGP NGPITVGLET AQALWLEIFC SFVFLFASIW MAYDHRQAKA
251 LGLVTVLSIV GIVLGLLVFI STTVTAKKGY AGAGMNPARC FGAAVVRGGH
301 LWDGHWIFWV GPTIACVAFY VYTKIIPPQH FHADGYKYDF IGVVKASFGL
351 HV
```

Supplementary Figure 2. Sequence coverage of *Nb*XIP1;1α obtained with the Orbitrap.

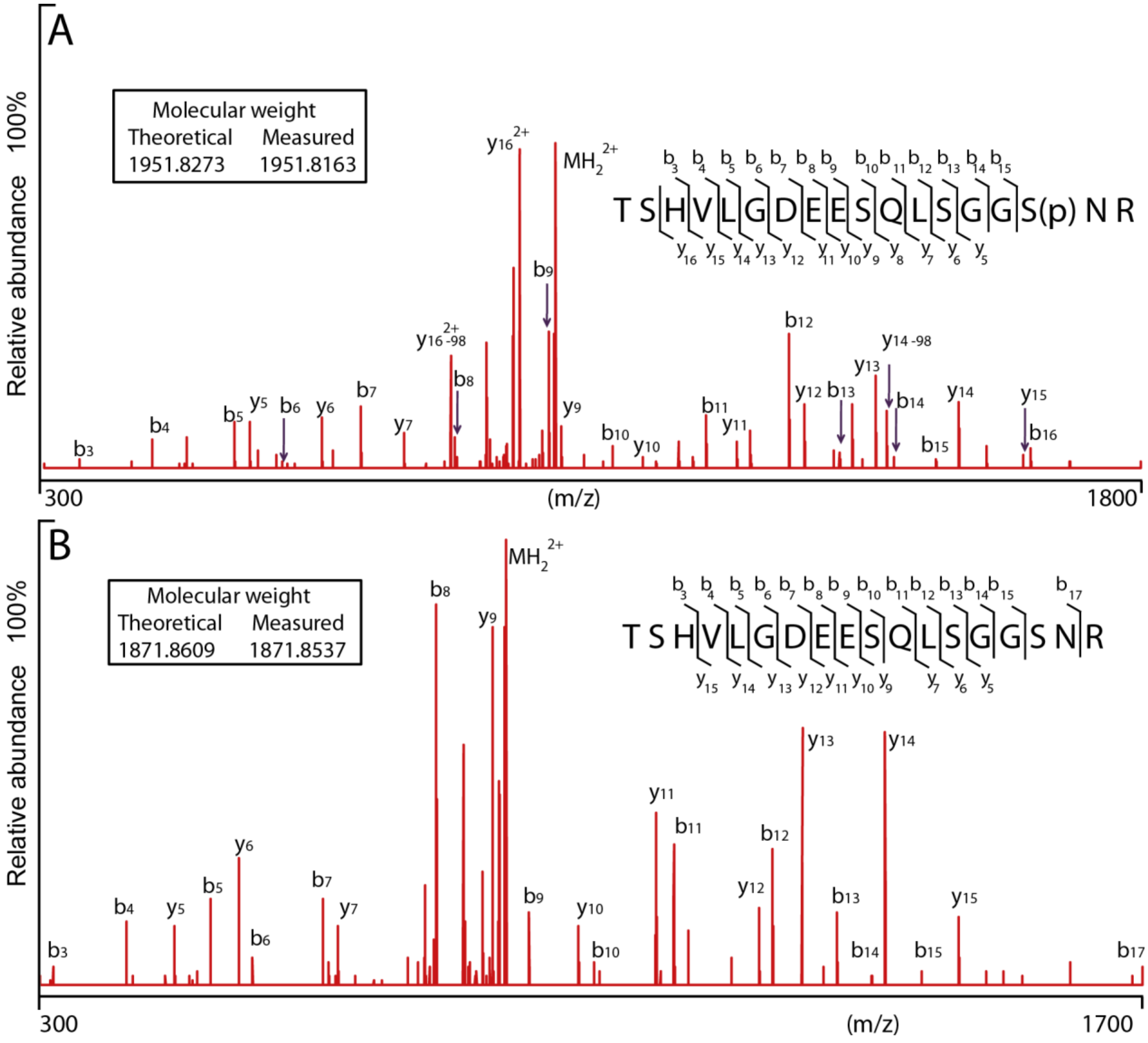

Supplementary Figure 3. Example of identification of phosphorylated peptide. Representative tandem mass spectra of the double charged (TSHVLGDEESQLSGGSNR) peptides from *Nb*XIP1;1α protein. The assigned fragmentation pattern consisting of b- and y-ions confirms the identity of the peptides and the presence of a phospho group on the S47. Some fragment ions are denoted in (**A**) as -98 Da, which corresponds to the loss of phosphoric acid. **(A)**. MSMS of the phosphorylated TSHVLGDEESQLSGGSNR peptide with S47 phosphorylated. **(B)**. MSMS of the TSHVLGDEESQLSGGSNR peptide where S47 is found without modification.

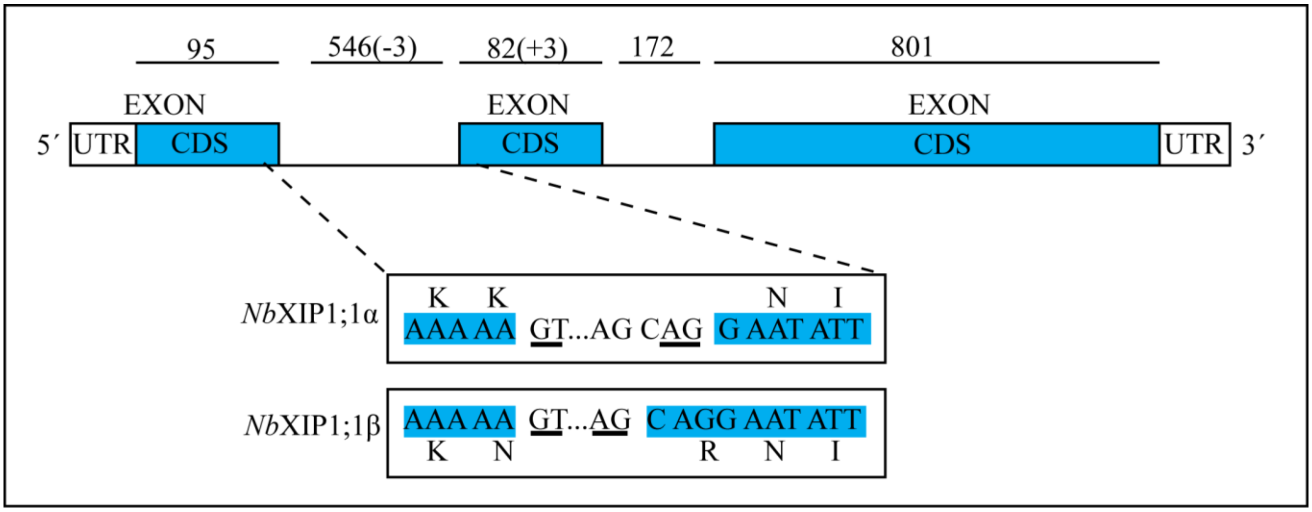

Supplementary Figure 4. *Nb*XIP1;1 gene structure and alternative splicing sites. A representation of the genomic DNA of *Nb*XIP1;1. Horizontal bars and gaps represent exons and introns respectively. The lengths of the exon and intron nucleotides are indicated. The first exon-intron-exon borders are enlarged showing the alternative splicing sites (GT …(AGC)AG and GT…AG) in *Nb*XIP1;1, resulting in the α and β splice variants, respectively. The nucleotide and protein sequences at the splice site of the two *Nb*XIP1;1 splice variants are displayed and the canonical dinucleotide intron borders are underlined.

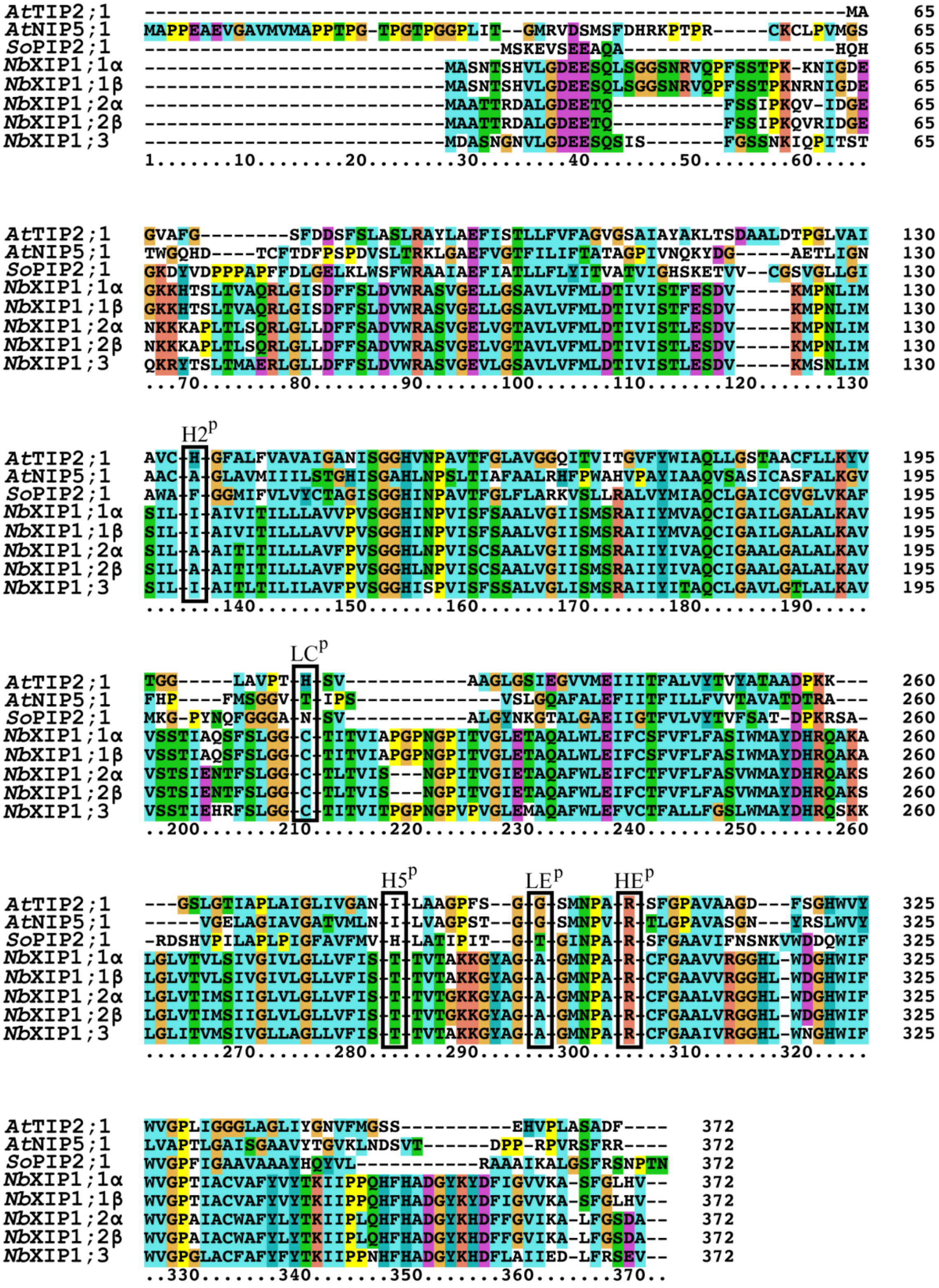

Supplementary Figure 5. Complete alignment of the five *Nb*XIP proteins, *At*NIP5;1 and the two plant AQPs with a solved structure (*So*PIP2;1 and *At*TIP2;1). The ar/R filter amino acid residues are shown in black boxes.

Supplementary Table 1. Detected peptides. Cutoffs: Mascot score ≥ 25, peptide mass tolerance ± 6 ppm.

Number	Sequence	Mascot score	Charge	Peptide mass tolerance (ppm)	Experimental Weight	Theoretical Weight
11 - 27	HHDSNGIPTENLYFQGM	81	3+	-2.43	1958.8533	1958.8581
24 - 49	FQGMSASNTSHVLGDEESQLSGGSNR	131	3+	-0.97	2694.1914	2694.1940
26 - 49	GMSASNTSHVLGDEESQLSGGSNR	87	3+	-4.08	2419.0571	2419.0670
26 - 49	GMSASNTSHVLGDEES(p)QLSGGSNR	95	3+	-3.48	2499.0246	2499.0333
28 - 49	SASNTSHVLGDEESQLSGGSNR	157	2+	-5.19	2230.9934	2231.0050
28 - 49	SASNTSHVLGDEESQLS(p)GGSNR	92	3+	-4.38	2310.9612	2310.9714
28 - 49	SASNTSHVLGDEES(p)QLSGGSNR	97	3+	-2.99	2310.9645	2310.9714
28 - 58	SASNTSHVLGDEESQLS(p)GGS(p)NRVQPFSSTPK	60	4+	-3.57	3362.4333	3362.4453
32 - 49	TSHVLGDEESQLSGGSNR	134	2+	-3.89	1871.8537	1871.8609
32 - 49	TSHVLGDEESQLSGGS(p)NR	82	2+	-5.62	1951.8163	1951.8273

32 – 58	TSHVLGDEESQLS(p)GGS(p)NRVQPFSSTPK	66	3+	-1.29	3003.2974	3003.3012
50 – 58	VQPFSSTPK	44	2+	-5.31	989.5129	989.5182
50 - 58	VQPFSST(p)PK	42	2+	-4.56	1069.4796	1069.4845
50 - 59	VQPFSST(p)PKK	30	2+	-4.79	1197.5737	1197.5795
50 - 66	VQPFSST(p)PKKNIGDEGK	41	2+	-3.70	1910.9068	1910.9139
50 - 67	VQPFSST(p)PKKNIGDEGKK	31	2+	-4.12	2039.0004	2039.0088
59 - 66	KNIGDEGK	50	2+	-3.19	859.4372	859.4399
59 - 67	KNIGDEGKK	55	2+	-3.11	987.5318	987.5349
59 - 76	KNIGDEGKKHTSLTVAQR	82	2+	-5.72	1981.0591	1981.0705
67 - 76	KHTSLTVAQR	42	2+	-4.45	1139.6360	1139.6411
68 - 76	HTSLTVAQR	44	2+	-3.99	1011.5421	1011.5461
68 - 76	HTS(p)LTVAQR	63	2+	-4.47	1091.5076	1091.5125
77 - 89	LGISDFFSLDVWR	107	2+	-3.28	1553.7827	1553.7878
90 - 103	ASVGELLGSAVLVF	55	2+	-2.89	1360.7563	1360.7602
104 - 118	MLDTIVISTFESDVK	96	2+	-2.51	1696.8551	1696.8593
174 - 185	CIGAILGALALK	90	2+	-2.60	1198.7076	1198.7107
186 - 196	AVVSSTIAQSF	78	2+	-3.26	1108.5728	1108.5764

186 - 201	AVVSSTIAQSFSLGGC	56	2+	-3.36	1582.7608	1582.7661
197 - 211	SLGGCTITVIAPGPN	50	2+	-2.46	1455.7356	1455.7392
212 - 222	GPITVGLETAQ	44	2+	-3.13	1084.5730	1084.5764
278 - 289	KGYAGAGMNPAR	84	2+	-4.58	1191.5764	1191.5818
279 - 289	GYAGAGMNPAR	71	2+	-2.84	1063.4839	1063.4869
290 - 297	CFGAAVVR	60	2+	-2.80	878.4408	878.4433
298 - 305	GGHLWDGH	48	2+	-3.68	877.3799	877.3831
298 - 313	GGHLWDGHWIFWVGPT	26	3+	-3.00	1863.8790	1863.8846
316 - 324	CVAFYVYTK	72	2+	-3.66	1149.5487	1149.5529
325 - 337	IIPPQHFHADGYK	29	2+	-3.19	1521.7617	1521.7728
324 - 345	IIPPQHFHADGYKYDFIGVVK	25	2+	-1.15	2443.2660	2443.2689
332 - 345	HADGYKYDFIGVVK	82	2+	-1.45	1610.8070	1610.8093
333 - 345	ADGYKYDFIGVVK	90	2+	-0.38	1473.7498	1473.7504
338 - 345	YDFIGVVK	60	2+	-3.25	939.5035	939.5066
346 - 352	ASFGLHV	50	2+	-1.96	729.3795	729.3810
